# Nanoscale pathogens treated with nanomaterial-like peptides: a platform technology appropriate for future pandemics

**DOI:** 10.2217/nnm-2020-0447

**Published:** 2021-05-14

**Authors:** Alaa F Nahhas, Alrayan F Nahhas, Thomas J Webster

**Affiliations:** ^1^Biochemistry Department, College of Science, King Abdulaziz University, Jeddah 21589, KSA; ^2^Department of Chemical Engineering, College of Engineering, Northeastern University, Boston, MA 02115, USA

**Keywords:** antiviral peptides, COVID-19, nanomaterials, nanoparticles, nanotechnology

## Abstract

Viral infections are historically very difficult to treat. Although imperfect and time-consuming to develop, we do have some conventional vaccine and therapeutic approaches to stop viral spreading. Most importantly, all of this takes significant time while viruses continue to wreak havoc on our healthcare system. Furthermore, viral infections are accompanied by a weakened immune system which is often overlooked in antiviral drug strategies and requires additional drug development. In this review, for the first time, we touch on some promising alternative approaches to treat viral infections, specifically those focused on the use of platform nanomaterials with antiviral peptides. In doing so, this review presents a timely discussion of how we need to change our old way of treating viruses into one that can quickly meet the demands of COVID-19, as well as future pandemic-causing viruses, which will come.

## Graphical abstract

B and D reproduced with permission from [1] © 2020 Wrapp *et al.*, licensed with CC BY 4.0.

## Virus pandemic & timescales for vaccine development

All viruses are nanometer-scale pathogens and some have potentially high mortality rates. For example, at the time of writing, the world is experiencing the SARS-CoV-2 pandemic (COVID-19), which has already caused more than 2.5 million deaths. The influenza virus is the most common virus that affects millions of people worldwide every year, mainly during the winter months due to weakened immune systems. Other viruses, such as other strains of the flu, hepatitis C, smallpox, HIV, measles, herpes, SARS, Middle East respiratory syndrome (MERS), avian influenza and the respiratory syncytial virus all have also caused significant healthcare problems. Symptoms of these viral infections can be mild to very severe and in some cases, infection with such viruses can certainly lead to death.

Table 1 shows the global comparison of deaths caused by these viral infections according to the WHO and CDC [2,3]. The high rate of mortality in some countries might be due to problems in vaccine delivery, the high cost of the vaccine, or the manner in which the medicine needs to be stored. Fortunately, most viral diseases already have a vaccine used to protect people; however, for each, precious time was spent for their development, costing human life. In fact, some vaccines took over a millennium to be discovered, as was the case for smallpox [4]. Fortunately, this is not the case for all vaccine development, but still the time spent on conventional vaccine development is simply too long. As just another example, it took 5 years to develop the measles vaccine, which is considered the fastest vaccine to be developed at a time when there were high technical limitations; in reality, it took a decade for the measles vaccine to be licensed and become readily available [5]. The poliovirus vaccine took 46 years to be developed [6]. Hopefully, this will not be the case with the COVID-19 vaccine, but at the time of writing, we are already well into the second year of this pandemic, and while several vaccines have been approved by various regulatory agencies, the globe is still waiting for wide vaccine distribution. SARS-CoV-2 vaccines from companies like Pfizer, Moderna and AstraZeneca have all been approved but took a significant amount of time to develop while the world shut down and people died [7]. However, when and if viral infections occur, human life cannot always be saved, particularly if the infection reaches an advanced stage. This is especially problematic when a vaccine is not available or takes a long time to develop [8].

**Table 1.** The epidemiological comparison of death between different viral infection diseases.

Viruses	Deaths (globally)	Ref.
Influenza	∼290,000–650,000	[9]
Hepatitis C	399,000	[10]
Smallpox	None	[11]
HIV	∼690,000	[12]
Measles	>140,000 since 2018	[13]
Herpes	None	[14]
SARS	None since 2003	[15]
MERS	858 since 2019	[16]
Avian flu	616 since 2013	[17]
Respiratory syncytial virus	∼59,600	[18]
COVID-19	Currently increasing daily; not under control	[2]

COVID-19: Coronavirus 2019; MERS: Middle East respiratory syndrome.

## Nanomaterial approaches

Nanoparticles as therapeutic agents are widely used to improve the treatment of numerous diseases like cancer, neurological diseases and infectious diseases [19]. Liposomes were some of the first nanoparticles to be synthesized and used for drug delivery, showing much promise [20]. Liposomes self-assemble into spherical vesicles consisting of bilayer lipid-like structures in aqueous solution [21]. These spherical vesicles have the ability to act as carriers to entrap hydrophilic drugs, helping drug stability and half-life [21,22]. For example, Epaxal is a liposome-based vaccine used to treat hepatitis A virus, with a particle size of 150 nm [23]. Polymeric nanoparticles have also been used to improve the efficacy of drugs by encapsulating hydrophilic or hydrophobic drugs or macromolecules [24]. Dendrimers are an example of polymeric nanoparticles that can be composed of synthetic or natural elements (e.g., amino acids) and their sizes depend on their functionalized surface [25]. They have an outer layer and an interior layer with a center cavity [26]. Small molecules or drugs can be loaded into such cavities [27]. The outer layer can be formulated with functional groups to targeting attachment sites [28]. Virus-like particles (VLPs) are particles self-assembled with the proteins of many viruses, with sizes ranging from 22 to 150 nm. These particles are noninfectious until they are incorporated with genetic material [29]. VLPs might be used as a vaccine against diseases due to their effectiveness in stimulating immune responses [30]. The human hepatitis B vaccine is an example of a licensed recombinant VLP from yeast [31]. Conjugation of theses nanomaterials to peptides improves their properties for various medical applications, as shown in Table 2.

**Table 2.** Advantages of the conjugation of nanomaterials with peptides.

Nanomaterials	Examples of peptides conjugated with drugs alone in drug delivery	Advantages of conjugating nanoparticles with peptides	Ref.
Gold nanoparticles	IIEGLYGLYASNLEU SER ALA, GLYVALALAIIETHRMETLYS, HISSERTHRPROSER SER PRO, ASNASPLEUMETASNARGALA, and ASPSERSERLEUPHEALALEU	Gold nanoparticles provide for extended half-lives to 21–22.3 h Favorable physicochemical properties Safety properties	[32]
Dendrimers (e.g., PAMAM)	Peptide (RGD) loaded with siRNA	Binding property enhancements with a receptor Enhancements in selectivity, stability and solubility	[33]
Liposomes	Tumor-homing peptide (internalizing RGD) and its sequence is CRGDKRGPDC	Enhancement in efficacy, longer permeability and retention effect	[34–36]

PAMAM: Poly(amidoamine).

**Figure 1.**
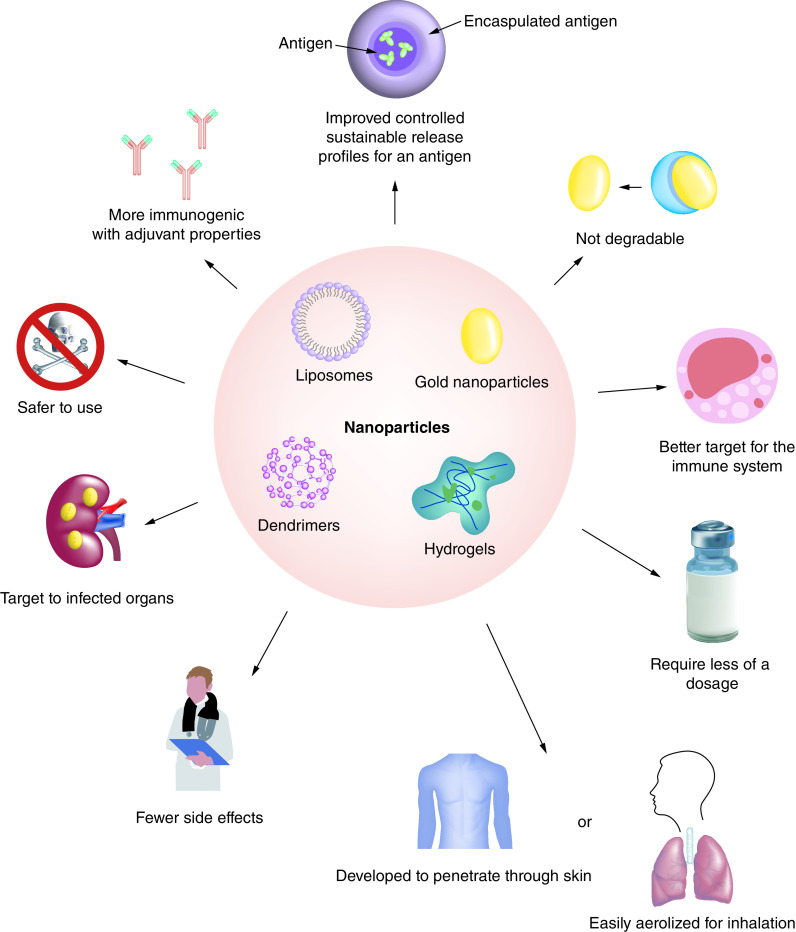
The advantages of using nanoparticles as a delivery tool for a vaccine over conventional adjuvants. These nanoparticles exhibit specific features that make them an attractive tool in medicine. Liposomes, with their hollow center and bounded by a lipid membrane, exhibit colloidal properties. Gold nanoparticles can be used as nanocages, nanoshells or nanorods that exhibit different ranges of particle sizes (0.001–8 μm). Dendrimers are polymers with uniform structures with monodispersed characteristics in drug delivery, self-assembling into vesicles and micelles, and have charges on their surfaces to help formulate them into different shapes. Hydrogels are polymeric networks exhibiting colloidal gel structures which absorb large amounts of water.

Due to the urgent need to find additional vaccines for COVID-19 to quickly save human lives (and for all future viruses yet unknown), it is clear that we need alternative approaches [37]. Incorporating nanomedicine into vaccine development will accelerate such development for many reasons including that it may provide a platform technology in which nanomaterials can be easily modified based on viral mutations and/or newly emergent viruses to attach to and kill viruses. Further, there are many advantages of using nanoparticles as a delivery tool for a vaccine over conventional adjuvants. They have fewer side effects, require lower dosage, can better target the immune system, have improved controllable and sustainable release profiles for an antigen, are safer to use, are more immunogenic with adjuvant properties (directly targeting the virus without the need to stimulate the host immune response), can be easily aerosolized for inhalation or developed to penetrate through skin, and can also target infected organs (which can change per virus exposure route) as shown in Figure 1 [38]. Because of all of these advantages, nanoparticles have recently become a great tool for drug delivery in the field of vaccinology [39]. Specific examples include chitosan, a natural nanoparticle with an average size of 400 nm that has been formulated with a vaccine against swine influenza, enabling cellular response induction [40]. Hyaluronic acid is another example that has also been used for intranasal vaccination to induce IgA against influenza [41]. Silver nanoparticles and dendrimers also exhibit potential antiviral activity [42,43]. In 2018 Du *et al*. designed silver nanoclusters with an average size ranging from 2.5 to 5.5 nm, which exhibited antiviral activity against the porcine epidemic diarrhea virus, considered a model of coronavirus [44]. These nanomaterials were selected due to their unique properties of high surface area and surface charge for better attachment [45]. Further, their smaller size allows the nanoparticles to easily penetrate the blood–brain barrier and the blood–testis barrier for viruses that reside in such anatomical locations, overcoming one of the major limitations of traditional drugs [46]. Loading antigens into nanoparticles might induce IFN-γ or IL-4 cytokines that contribute to the immune response [47]. This is a main point when designing nanoparticles, because they can avoid immune system clearance to deliver a biomolecule more efficiently than conventional approaches. These nanoparticles can be engineered with drugs to target organs, tissues or even individual cells with improved delivery and retention times [45]. Moreover, nanoparticles composed of natural and biodegradable chemistries (e.g., iron oxide or selenium) can simultaneously boost a patient’s immune system while deactivating a virus.

## Targeting a virus

**Figure 2.**
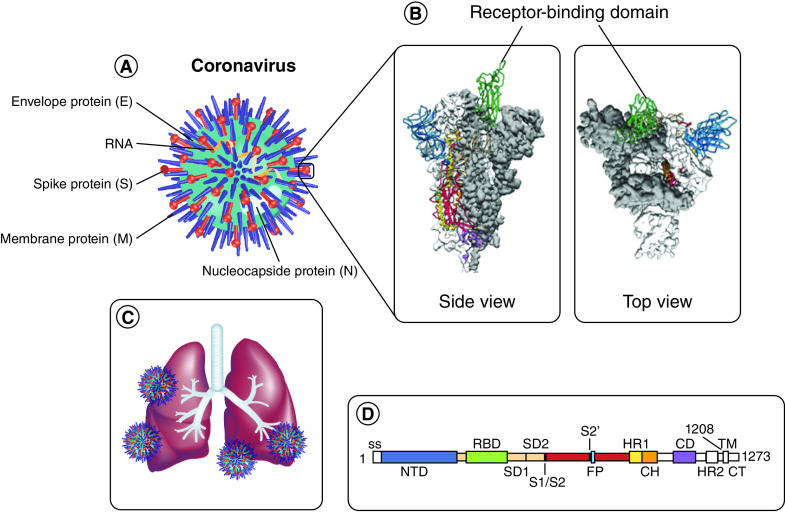
The structure of SARS-CoV-2. **(A)** Representative structure of the virus. **(B)** Side and top view of the spike protein of the virus. **(C)** Area where the virus is mainly infected. **(D)** Classification of the viral domains. CD: Connector domain; CH: Central helix; CT: Cytoplasmic tail; FP: Fusion peptide; HR1: Heptad repeat 1; HR2: Heptad repeat 2; S2′: Protease cleavage site; SS: Signal sequence; TM: Transmembrane domain. Reproduced with permission from [1] © Wrapp *et al.*, licensed with CC BY 4.0 (2020).

Targeting and killing a virus requires discovering an antiviral drug that does not have significant secondary effects in humans. Some viral processes, such as the way in which they enter the host cell to begin the replication process, are still largely unknown. For COVID-19, this is a major global research effort because understanding such an exact mechanism can help in developing a therapy. Figure 2 shows the first cryogenic electron microscopy image of the spike protein on COVID-19 [1]. From this structure, that protein can be targeted when designing an anti-COVID drug. According to the SARS-CoV-2 docking sever website [48], these proteins possess the spike receptor-binding domain (RBD), the main protease, a spike monomer or trimer, E protein as a monomer or ion channel, RNA-dependent RNA polymerase, helicase NCB site, Nsp14, Nsp15 (endoribonuclease), Nsp16 (2′-O-MTase) and Nsp10. One of the most suspected target proteins for SARS-CoV-2 is the 3C-like proteinase [49]. This enzyme plays a vital role in cutting the viral polypeptide into small pieces and helps the virus replicate.

**Figure 3.**
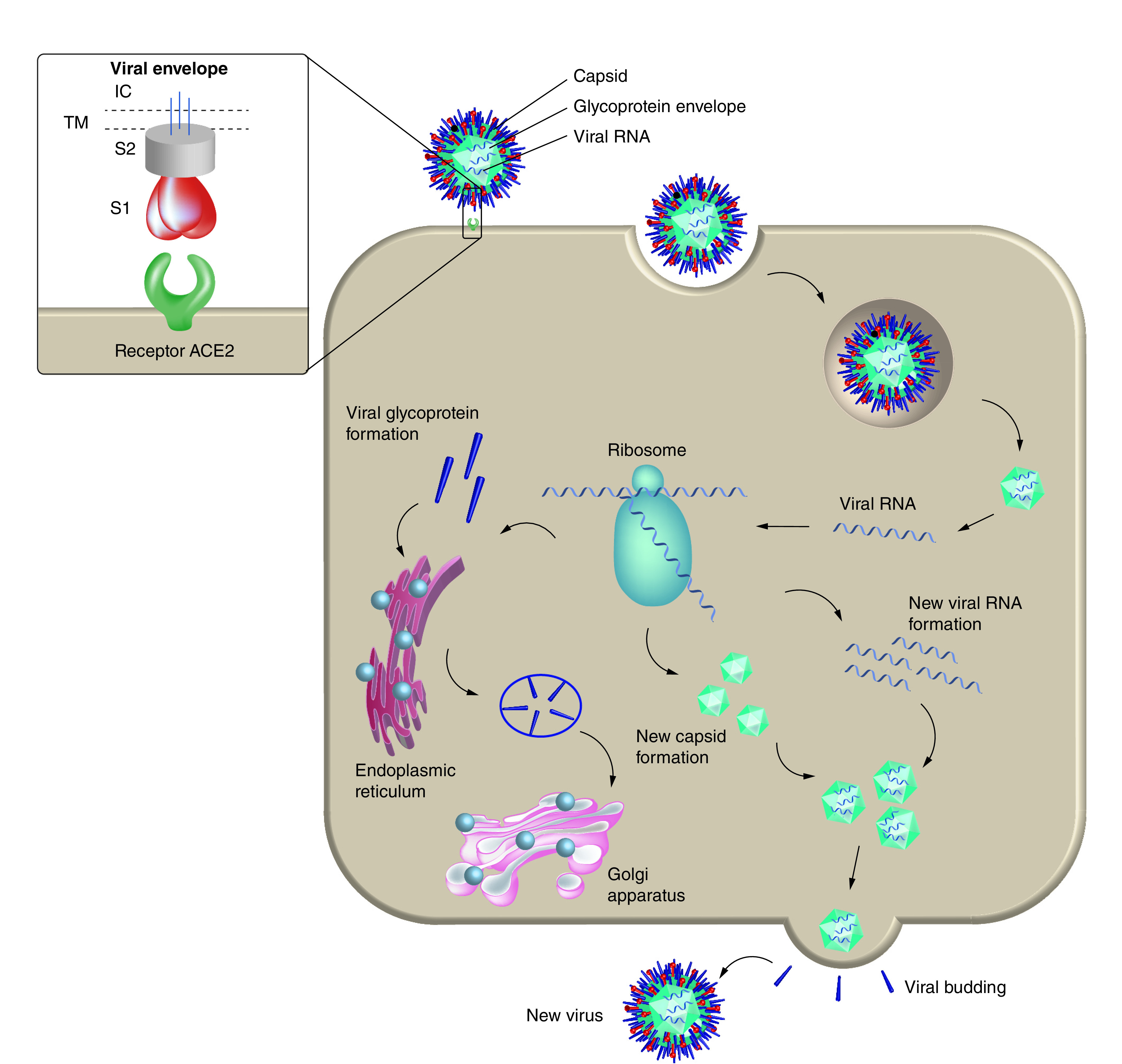
A representative schematic of the viral life cycle. The viral life cycle can be summarized as follows: virus binds to the cell host through a receptor binding site, fuses into the host cell, releases its genome, synthesizes its protein, multiplies, assembles again from its building blocks and releases from the host cell to start the whole process again. The small box shows the receptor-binding subunit S1, the membrane-fusion subunit S2, the IC tail, the TM anchor and the viral envelope. IC: Intracellular; TM: Transmembrane.

When designing an antiviral drug, two mechanisms can be considered: targeting the virus itself or targeting the host cell biological functions to keep the virus from replicating. Targeting the virus itself is done by inhibiting the biological function of the virus, such as its proteins or replication enzymes [50]. In contrast, designing a drug that inhibits the host biological activity can be achieved by inhibiting the host’s cellular factors and proteins that are important for the viral life cycle [51,52]. Figure 3 shows the life cycle of the virus.

The mechanism that describes the invasion of human cells by viruses is a key point that needs to be addressed when designing an antiviral drug. As an example, we cite the case of SARS-CoV, which first appeared in 2002. The mechanism by which the virus invaded human cells was unknown, which led to a pandemic. The SARS vaccine took about 20 months to reach clinical trials. Two potential SARS vaccines were evaluated in a Phase I clinical trial; however, these vaccines were not tested further because SARS as a virus disappeared by itself. The first SARS-CoV vaccine was discovered by Sinovac Biotech and used an inactivated whole virus vaccine [53]. That type of vaccine did not show any immunogenic problems and is safe [53]. The second vaccine was developed using a recombinant plasmid DNA vaccine encoding the SARS spike glycoprotein [54]. These two vaccines were not the only ones developed then; many more were developed without any further studies on humans. For COVID-19, a similar effort is ongoing, even much faster. According to the coronavirus vaccine tracker website, there are 21 vaccines in large-scale efficacy tests in Phase III; however, just six vaccines have been approved for limited use in some countries [55]. Some of these vaccines show good efficacy; for example, Moderna’s effort, which uses a lipid nanoparticle, showed 94.5 % effectiveness against the virus, while the Pfizer and BioNTech vaccines were 95% effective and have been approved for use in some countries (e.g., New Zealand, Switzerland, Bahrain and Saudi Arabia).

Researchers are constantly looking to unveil the process by which SARS-CoV-2 penetrates a human cell to replicate, obviously looking for a mechanism to stop it. SARS-CoV-2, which is related to the coronavirus family, shows the ability to bind to ACE2 on the surface of a host cell through its spike protein that contains two domains: an S1 RBD and the S2 fusion domain [56,57]. The S1 RBD is the first part of the virus that binds to the ACE2 receptor, then its binding facilitates the S2 domain to fuse to the host cell. The proteases Furin and TMPRSS2 help to cleave the S1 and S2 domains from each other [58,59]. The S1 subunit remains bound to ACE2; however, the S2 subunit fuses the viral capsid and helps viral entry. As a result, any cell that expresses ACE2 is a target for the virus [60].

## Antiviral peptides

Nanomaterials can also be easily functionalized to attach to viruses. For example, peptides, as a type of nanomaterial, consist of a short sequence of amino acids as building blocks and are much smaller in size and molecular weight than proteins. A few peptides self-assemble into unique well-organized structures that give them unique functions, and that is the main concern when designing them [61]. Peptide hormones, the most widely used therapeutic peptides, are hormones consistent with natural amino acid chains [62]. Insulin is the most useful injectable peptide medicine for treating diabetes type II [63]. Peptides are also excellent candidates for disrupting virus function. Peptides have shown excellent activity not just against bacteria and for promoting tissue growth, but also against viruses. Supplementary Table 1 shows some examples of the applications of peptides in the medical field and their associated activities. The ability of peptides to penetrate cell membranes or attach to viruses and even cross their lipid membrane envelope can further increase their usage in the drug design and drug delivery industry. Peptides are widely used in the medical field due to their attractive highly controllable structural properties, such as their topology or composition and other chemical properties, which allow them to form specific secondary structures and play a vital role as antiviral drugs [64]. Peptides as therapeutic agents have demonstrated lower toxicity and more specificity of action compared with other drugs; however, there are some limitations to their use [65]. Peptides are subject to degradation by enzymes and thus may have short half-lives. One way to overcome this issue is by using peptide carriers (such as nanoparticles) that protect them from the environment to reach their destination safely and reduce the immune response [66]. Examples of these nanocarriers include micelles, polymeric nanoparticles and liposomes [67]. Another way to lengthen the half-life of peptides is to identify the enzyme molecular cleavage sites on the peptides and replace them with relevant amino acids. We can also overcome this problem by designing cyclic peptides or peptides that contain unnatural amino acids, to make their recognition by enzymes more difficult [68–70].

Peptides have been used in nanomedicine for over a decade due to their promising properties that can be exploited in the biomedical field and their biocompatibility, remaining nontoxic to surrounding healthy tissues or cells [71]. Peptides have physical proprieties that make them an attractive tool to be used in medicine. For example, Badani *et al.* have shown that the hydrophobicity of peptides provides an excellent antiviral effect [72,73]. Due to that property, some peptides showed great inhibitory activity against the entry of enveloped viruses, and these peptides are called antiviral peptides [64,74]. Such peptides can inhibit viral entry to the host cells due to their hydrophobicity. acting as competitive inhibitors by interfering with binding [75]. The synthetic linear peptides SA-35 (Met-Ile-Thr-His-Gly-Cys-Tyr-Thr-Arg-Thr-Arg-His-Lys-His-Lys-Leu-Lys-Lys-Thr-Leu) and dendrimer LTP ((((Arg)_2_Lys)_2_Lys)_2_Lys-Ala-Cys) peptides have been shown to exhibit excellent antiviral activity against the respiratory syncytial virus, which causes a dangerous disease mainly affecting children [76]. The main characteristic of these two peptides is the evenly positive charge distribution with low amphipathic amino acid residues. These peptides were synthesized according to the specific bioactive cationic and helical regions of the structure of the respiratory syncytial virus cellular receptor, nucleolin [76].

López-Martínez *et al.* designed nine peptides based on the conserved region on the viral spike protein, hemagglutinin [77]. The nine peptides showed great inhibition against the human swan and avian influenza strains at a concentration of 20–74μM. This inhibition rate might be due to the peptide interference between the viral spike protein and the host cell surface. Another group of scientists focused on a different region of the viral spike protein, neuraminidase, which helps the virus in the replication process [78]. They designed an octapeptide (errKPAQP) that acts as a neuraminidase inhibitor; it reduced the rate of virus replication at a concentration of inhibition as low as 4.25μM [78]. Hemagglutinin and neuraminidase are the two main glycoproteins responsible for viral fusion that are targeted when designing antiviral peptides [78,79]. Matsubara *et al.* synthesized two pentapeptides, c01(GWWYKGRARPVSAVA) and c03 (RAVWRHSVATPSHSV), according to the specific binding of hemagglutinin to the Neu5Acα2–3Gal-containing ganglioside GM3 on the cell surface receptor [80]. These two peptides were then acylated with a C18 group to help their assembly and multivalent binding to the virus. Both of them exhibited influenza virus binding to the cells [80]. Six potential peptides recently reached preclinical trails: SBP1 (IEEQAKTFLDKFNHEAEDLFYQS) [81], EK1C4 (SLDQINVTFLDLEYEMKKLEEAIKKLEESYIDLKELGSGSG-PEG4-Chol) [82], P9R (NGAICWGPCPTAFRQIGNCGRFRVRCCRI) [83], QS1 (Ac-Abu-Tle-Leu-Gln-VS) [84], VIR251 (Ac-hTyr-Dap-Gly-Gly-VME) [85] and VIR250 (Ac-Abu(Bth)-Dap-Gly-Gly-VME) [85]. Multivalent peptides or nanomaterials might be a new avenue for the treatment of SARS-CoV-2, as suggested by Tabish *et al.* [86]. It has been reported that 6-sialyllactose-polyamidoamine has multivalent functions of acting as an anti-influenza inhibitor and an inhibitor of H1N1 infection [87]. Monoclonal antibodies can inactivate the spike protein and inhibit virus entry [88]. That strategy has been researched since the beginning of the pandemic and will be investigated further in future pandemics.

Recently, Webster *et al*. designed self-assembled molecules such as Twin Based Linkers functionalized with amphiphilic peptide nanoparticles that were more strongly attracted to SARS-CoV-2 than the peptides alone using computational models (unpublished data). Such amphiphilic peptides exhibited antibacterial activity as well [89]. Nanoparticles also play an important role when they are conjugated with nonpeptide antiviral compounds. For example, a fragment of the potent HIV inhibitor TAK-779 (SDC-1721) alone did not exhibit antiviral activity against HIV; however, when conjugating SDC-1721 with 2-nm gold nanoparticles, excellent inhibition of TAK-779 was observed [90]. Solid lipid nanoparticles are another example of the enhanced bioavailability of highly lipophilic drugs, including Efavirenz, which is used to treat HIV [91]. From these results, it is clear that peptides might be good candidates as antiviral drugs and that attaching them to nanocarriers can make them even better. Table 3 shows examples of some of the more popular antiviral peptides.

**Table 3.** Examples of antiviral peptides and their regions acting on a virus.

Peptides	Specific acting region	Source	Ref.
N1LB-HA, N2LB-HA, N3LB-HA, C1LB-HA, C2LB-HA, C3LB-HA, PHGB-1, PHGB-2, PHGB-3	Viral surface glycoproteins HA	Derived from highly conserved sequences of HA1 & HA2 subunits	[77]
errKPAQP	Viral surface glycoprotein neuraminidase	The specific sequence in the binding pocket of oseltamivir (a drug) in neuraminidase	[78]
c01 (GWWYKGRARPVSAVA) and c03 (RAVWRHSVATPSHSV)	Binding to sialylgalactose (Neu5Ac-Gal)	Specific recognition region of HA to the sialyloligosaccharides of glycoproteins on the cell surface receptor	[80]

HA: Hemagglutinin.

Recently, researchers have focused on peptide-based approaches to design a vaccine based on preliminary molecular dynamics computational simulation studies. Peptides have many advantages over small molecules when designing a drug. They can be synthesized easily with low cost and less toxicity. A peptide isolated from ACE2 was found to significantly block SARS-CoV-2, but its binding efficacy could be speculatively much higher when multiple nanocarrier linkages bind to the peptide [92]. For example, Mansoor *et al.* encapsulated poly(d,l-lactic-co-glycolide) nanoparticles to bovine parainfluenza 3 virus (BPI_3_V), which causes bovine respiratory disease, and found that by attachment to the nanoparticles, there was a significant enhancement in the mucosal BPI_3_V-specific IgA response [92]. Encapsulation of the virus with the nanoparticles would slow its release in the mucosa. One of the advantages of using nanoparticles is their ability to cross the mucosal barrier due to their small size (100–200 nm) [93]. Protease enzymes of the host cell break down the viral spike protein into two segments – the outer segment (S1) and the inner segment (S2) – as shown in Figure 2. Only the S2 segment enters the host cell and replicates using the viral genome [57,94], and thus the design of antiviral drugs focuses mainly on targeting this interaction. Pant *et al.* screened 300 peptide-like structures based on ligand–receptor interactions as SARS-CoV-2 main protease inhibitors and found that four of these peptides had strong binding affinity [95]. These four peptide-like structures are CHEMBL303543, CHEMBL206650, CHEMBL127888 and CHEMBL573507; they might be promising compounds but need further investigation. These four peptides were computationally synthesized and showed higher binding affinity [95].

There are many peptides that have been used as drugs in clinical trials or even researched in the market [63]. Lupron is a peptide-based drug used to treat prostate cancer and reached global sales worth more than US$2.3 billion in 2011 [96]. A group of glucagon-like peptide-1 (GLP-1) agonists that has been used to treat Type 2 diabetes mellitus generated more than US$2.6 billion globally in 2013 [97]. Of course, selecting the right peptide for a specific medical application can be accomplished using a specific active sequence of known proteins or by using high-throughput screening [98]. In this review, we summarize synthetic antiviral peptides designed particularly for treating SARS-CoV-2 and suggest their mechanism for fighting the novel virus.

Moreover, researchers have shown that some properties of these peptides can be used to target a specific step in the viral life cycle, such as the virus glycoprotein, the viral replication step or the viral polymerase [99]. Antiviral peptides not only can block any step of the viral life cycle, but can compete with the virus binding site of the host cell and inhibit virus invasion [100]. It is because these peptides have a powerful effect on various viral life stages that many researchers have concentrated their work on them. This has led to the development of antiviral peptide databases which are readily available online (http://crdd.osdd.net/servers/avpdb). In this review, we mention some of the most useful antiviral peptides against SARS-CoV-2.

Peptides are soft nanomaterials used mainly as an alternative tool to inhibit the growth of different types of bacteria and viruses as well as to increase tissue growth in regenerative medicine. Many antimicrobial peptides can also act as antiviral agents, for example against herpes simplex virus, and have been designed according to these mechanisms. Some examples of such antimicrobial peptides are listed in Supplementary Table 2.

### Coronavirus-related diseases & antiviral peptides

According to a Harvard study, COVID-19 first appeared in August 2019 in Wuhan City, China [101]. It is the most recent coronavirus pandemic. The reason for the appearance of this disease is still unknown; however, researchers suggested that the virus was caused by a bat because more than 93.1% of the spike genes of SARS-CoV-2 share the same sequence as coronavirus RaTG13 [102]. This virus belongs to the Coronaviridae family, which mainly affects the human respiratory tract and has six classes: HCoV-229E, HCoV-OC43, HCoV-NL63, HCoV-HKU1, SARS-CoV and MERS-CoV [103,104]. These viruses affect different parts of the respiratory tract; for example, two of these classes, HCoV-229E and HCoV-OC43, affect the upper and middle parts of the respiratory tract. Infection with these viruses causes the same symptoms as normal seasonal flu.

Continuing to design and test new or existing vaccines for this and other viruses yet to come is an urgent priority. Knowledge of the detailed composition of viruses will make it more likely that a vaccine will be designed. Antiviral peptides showed potential activity against MERS-CoV and SARS-CoV, which could provide an alternative approach to be tested against COVID-19 [105,106]. Some of these peptides exhibit no cytotoxicity and a low immunogenic profile, considered as the main concern when designing such peptides [107]. Zhau *et al.* designed a P9 peptide derived from mouse β-defensin-4 (mBD4) and found that it exhibited antiviral activity against multiple respiratory viruses, including H5N1, H1N1, SARS-CoV and MERS-CoV [106]. β-defensin is a cysteine-rich peptide that is secreted from cells of the respiratory, urinary, digestive and reproductive tracts [108,109] and acts as an inhibitor of viral entry into cells and viral replication [110]. The P9 peptide (NGAICWGPCPTAFRQIGNCGHFKVRCCKIR) is a specific sequence of mBD4 that can bind viral glycoproteins and enter host cells with the virus through endocytosis, and which inhibits the replication of viral RNA. It has an IC_50_ of 1.2 μg/ml, which is even lower than the synthetic mBD4 [106]. In 2013, Gao *et al*. designed an antiviral peptide P1 (LTQINTTLLDLTYEMLSLQQVVKALNESYIDLKEL) with an EC_50_ (the concentration of a therapeutic agent that gives a half-maximal response) of about 3.013μM against pseudo-typed MERS-CoV. Their design was based on a conserved sequence called heptad 2 (HR2) that was present on the viral spike protein [111,112]. In the following year, Lu *et al*. designed peptides and tested whether these peptides could bind together and form a six-helix bundle (6HB) that can compete with the virus fusion core structure [113]. The rationale when designing the peptides is that the heptad repeat 1 (HR1) and HR2 join and form a 6HB fusion core on the viral spike [113]. Both of these heptads contain three segments. This research group designated the 36-mer HR2 peptide (SLTQINTTLLDLTYEMLSLQQVVKALNESYIDLKEL) and found that such peptides were efficient at inhibiting MERS-CoV spread and syncytium formation [113]. The longer the peptides, the better their antiviral activities [114].

In 2017 Sun *et al*. followed the same steps as Lu and designed a peptide to block the 6HB fusion core formation [115]. They found that their MERS-5HB peptide, containing three HR1 and two HR2 segments, bound to an HR2-derived peptide with a strong affinity and inhibited pseudotyped MERS-CoV entry with an IC50 value of ∼1μM [115]. This led to the blocking of the formation of the fusion core. The same group designed a 229E-HR2P peptide based on the HR2 region of a spike protein in the HCoV-229E and found antiviral activity with an IC50 value of 1.7μM against a live HCoV-229E infection [116]. The most recent landmark paper of Xia *et al.* described a pan-coronavirus-based fusion inhibitor on the HR1 domain of the human coronavirus spike, EK1, which has great potential to be used for future pandemics [117]. These are all promising results that have led to the design and testing of more peptides for antiviral activity. Supplementary Figure 1 shows the chemical structures of some of these promising antiviral peptides.

**Figure 4.**
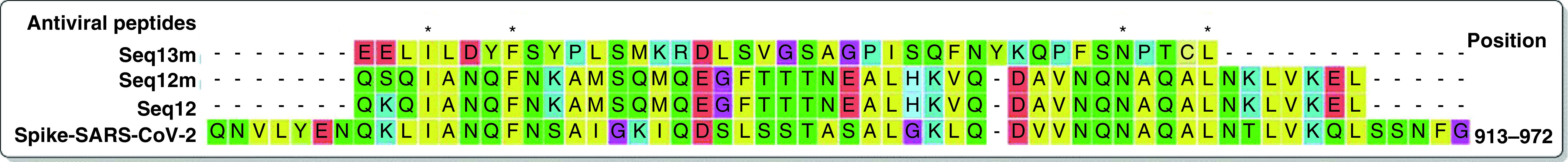
The spike glycoprotein mapping sequence of SARS-CoV-2 that aligned with the amino acid sequences of antiviral peptides, such as Seq12, Seq12m and Seq13m. These sequences have some common amino acids as represented with an asterisk. Reproduced with permission from [118], licensed with CC BY 4.0.

Dutta studied the genome sequences of SARS-CoV-2 and used some bioinformatic tools to understand the behavior of these amino acid sequences [118]. He studied the genetic sequences of the spike glycoproteins of the virus before starting to do any lab work. He aligned the sequences of those glycoproteins using the MEME suite and other software, such as antiviral peptide prediction servers (AVPred) to identify motifs [119]. Then he predicted three antiviral peptides whose amino acid sequences aligned with the viral spike glycoprotein, as shown in Figure 4. The three analogous antiviral peptides to the viral spike glycoproteins are Seq12, Seq12m and Seq13m [118]. The molecular dynamics process was used to study the physicochemical properties between these antiviral peptides and the binding pocket. Dutta found that the interaction of these peptides with the binding site was thermodynamically favorable and had efficient binding capacity. Further confirmation of these findings is needed to possibly find a vaccine for COVID-19.

Lan *et al*. identified the RBD of the spike glycoproteins for the ACE2 cell receptor [120]. Supplementary Table 3 shows the types of amino acids that constitute the RBD Additionally, it has been suggested that SARS-CoV can interact with some other mammalian cell surface receptors, such as aminopeptidase N and dipeptidyl peptidase-4 [121,122]. All of this information might provide a valuable clue for the next step in the process of finding a cure for the current pandemic.

Recently, the SARS-CoV-2 main protease structure was studied and crystallized as part of efforts to find inhibitors [123]. Zhang *et al.* cocrystallized the SARS-CoV-2 main protease structure with a peptide-like inhibitor, the α-ketoamide inhibitor [124]. These peptide-like inhibitors not only have antiviral activity but are useful in many other medical applications, such as for cancer, autoimmune disease [125] and diabetes [126]. Supplementary Figure 2 shows the chemical structures of these four inhibitors. A better understanding between the SARS-CoV-2 main protease composition and its properties can provide a clue on how to design antiviral drugs. The authors first did some docking and free energy calculations of the cocrystallized peptide-like inhibitors obtained from the Protein Data Bank, such as 6Y2F and 6W63, and then conducted molecular dynamic simulations using Schrödinger [127,128]. From this information, the SARS-CoV-2 binding site is known to have positively charged residues (Arg-188), two negatively charged residues (Glu-166 and Asp-187) and hydrophobic and hydrophilic residues [129]. Having obtained information about the binding site pocket, researchers tested some antiviral compounds that showed some interaction with SARS-CoV-2: saquinavir, nelfinavir, teniposide and apicidine [130,131]. All of these inhibitors recently entered clinical trials to be tested for treatment of COVID-19. Apicidine in particular showed inhibition against SARS-CoV-2 [130]. This result might be a starting point to relieve the COVID-19 pandemic.

### Peptides & the cytokine storm

One important item that requires strong consideration when designing antiviral drugs is to reduce the immune response, especially when dealing with the COVID-19 virus, due to the ‘cytokine storm’. The cytokine storm, the result of an overactive immune system that causes further complications and even death, has been observed with other viruses related to COVID-19, like influenza virus [132], SARS-CoV [133] and the avian H5N1 influenza virus [134]. The cytokine storm has been observed in patients with COVID-19 who have an uncontrolled inflammatory response or cytokine overproduction that can attack the body’s own cells as in an autoimmune disease [135].

**Figure 5.**
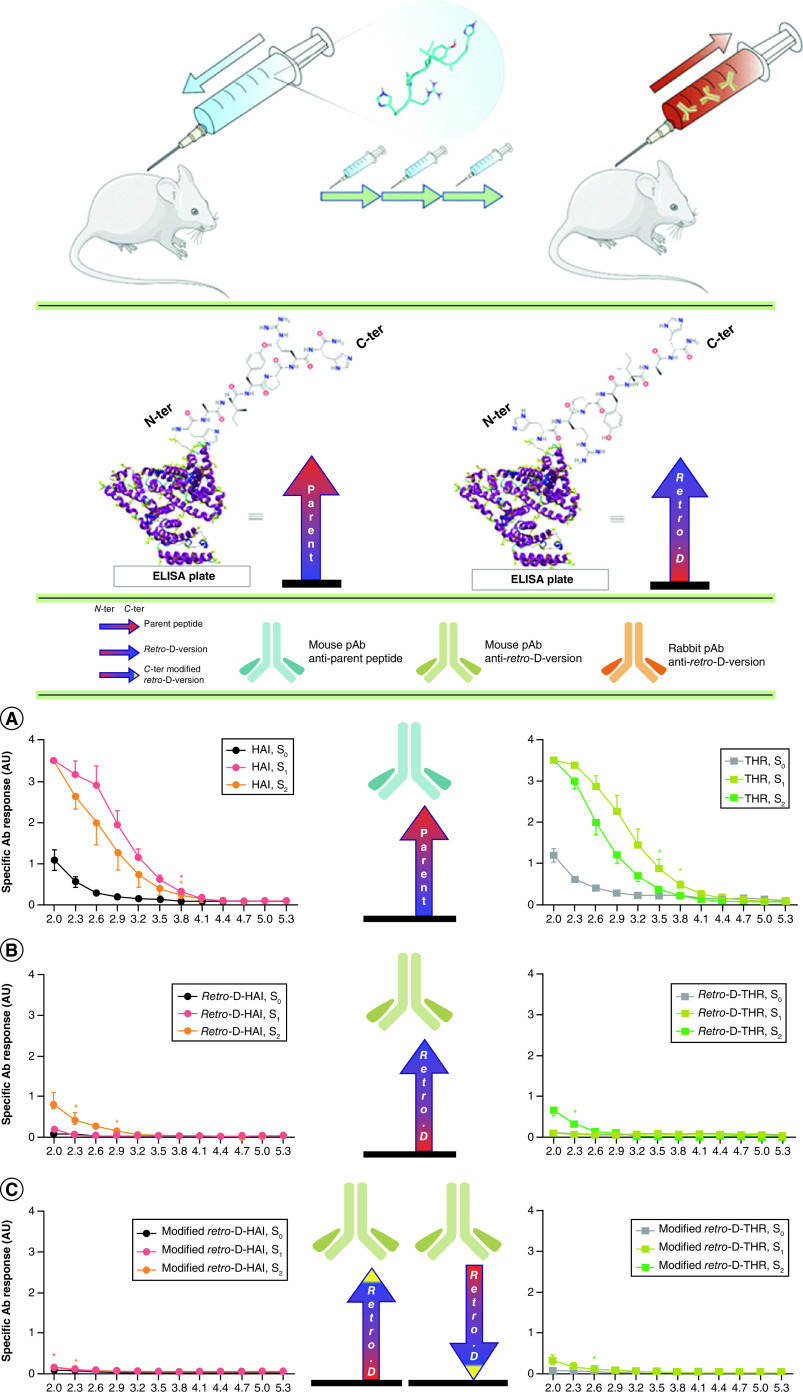
Retro-d-peptides induced little or no immune response compared with the l-natural peptides. The stereochemical inversion of l-natural peptides induced no immune response, as shown in **(B)** and **(C)** (retro-d-peptides) compared with **(A)** (l-peptides). This test was done by titration of the humoral response in mice using ELISA. **(A)** Serum anti-parent peptides (HAI and THR). **(B)** & **(C)** Serum anti-retro-d-peptides. Reproduced with permission from [142] © Arranz-Gibert *et al.*, licensed with CC BY 4.0 (2018).

Some approaches have been used when designing peptides to not elicit, or to minimally elicit, the immune response [136]; PEGylation is widely used for this purpose [137,138]. Retro-d-peptides containing retro-inverso/or enantio-isomers of l-amino acid-containing peptides, showed a low immune response and protease resistance [139,140]. Arranz-Gibert *et al*. designed the retro-d form of two families of peptide blood–brain barrier shuttles: the retro-d forms of the HAI (H_2_N-HAIYPRH-CONH_2_) and THR (H_2_N-THRPPMWSPVWP-CONH_2_) peptides [141,142]. The immunogenicity of HAI, retro-d-HAI, THR and retro-d-THR were compared using ELISA, as shown in Figure 5 [142]. A moderate immunogenicity response appeared with antibody titration with l-peptides but that signal that detected the antibody disappeared with the retro-d form-containing peptides. LL-37 is another example of the peptides mentioned earlier which exhibit antimicrobial activity without stimulating proinflammatory cytokines [143]. It can easily be imagined, and needs to be the focus of future applications, to combine some of the more exciting antiviral agents with the anti-inflammatory peptides mentioned herein into one nanocarrier. Only when this is accomplished will we have an approach that both is antiviral and can minimize the cytokine storm that results from COVID-19.

## Conclusion

The recent COVID-19 pandemic has led to more than 2.5 million deaths worldwide (a number which is still climbing) and has called upon us all to work on a treatment for this disease as soon as possible. This could save thousands and possibly millions of lives. One reason we do not have a current therapy or vaccine is that for COVID-19, a large number of genomic mutations exist, requiring scientists to go ‘back to the drawing board’ and almost start from scratch. This pandemic started in China and spread to the rest of the world, in a similar fashion to the spreading of the Ebola virus that started in the Democratic Republic of Congo, particularly in a village near the Ebola River in 2013, before it spread all over the world [144]. We clearly need a better strategy to develop treatments for viruses that operate under a faster timeline to avoid the loss of life during the processes of therapeutic or viral development.

The conventional treatment for all of these viruses (and those still to come) is still limited or insufficient. It also suffers from the fact that in some cases, side effects are severe. However, by knowing the composition of the binding site pocket of viruses like the SARS-CoV-2 main protease in depth, designing such an antiviral compound might be possible, in particular when one uses peptides. Peptides are easy to synthesize, have a low cost compared with small molecules and are excellent candidates in targeting specificity. Peptides such as LL-37 peptides show much higher activity against many viruses and have antiviral activity against influenza A [145,146]; the Mucroporin M1 peptide has antiviral activity against influenza H5N1 and SARS-CoV-2 [147,148]. Further, as this review shows, peptides can be attached to nanomaterials to provide a platform technology to treat viral infections and when combined with their anti-inflammatory properties to reduce the cytokine storm, can represent the future we badly need for this and the next virus pandemic.

## Future perspective

This review presents a timely discussion of how we need to change our old way of treating viruses into one that can quickly meet the demands of COVID-19 as well as future pandemic-causing viruses, which will come. It is clear from the COVID-19 pandemic that our approach to vaccinating against and developing therapies for viruses is antiquated and too slow, and needs to change significantly. Future research in this area is badly needed to develop general strategies focused on peptide design and nanomaterial delivery that can serve as a generalized approach for treating this and future pandemics. In doing so, we will have developed a platform technology that just needs to be simply adapted to each new virus that emerges or mutation that develops, with general safety regulatory approval. Only once we have developed platform technologies will we be better prepared for any viral infection – a lesson all too well learned in this pandemic.

**Executive summary**

**Virus pandemic & vaccine duration**

- One reason we do not have a current therapy or vaccine for COVID-19 is that a large number of genomic mutations exist, causing us to go ‘back to the drawing board’ and almost start from scratch.
- We clearly need a better strategy to develop treatments for viruses that operate under a faster timeline to avoid the loss of life in the processes of therapeutic or viral development.

**Nanomaterial approaches**

- By knowing the composition of the binding site pocket of viruses like the SARS-CoV-2 main protease in depth, designing such an antiviral compound might be possible, particularly when peptides are used.
- Peptides are easy to synthesize, have a low cost compared with small molecules and are excellent candidates in targeting specificity.

**Peptides & the cytokine storm**

- Further, as this review shows, peptides can be attached to nanomaterials to provide a platform technology to treat viral infections and when combined with their anti-inflammatory properties to reduce the cytokine storm, can represent the future we badly need for this and the next virus pandemic.
