# Supplementary figures and images for: Nanoscale pathogens treated with nanomaterial-like peptides: a platform technology appropriate for future pandemics
Source: Nanomedicine (Lond). 2021 May 14:10.2217/nnm-2020-0447. doi: 10.2217/nnm-2020-0447 (PMC8120868; doi:10.2217/nnm-2020-0447)

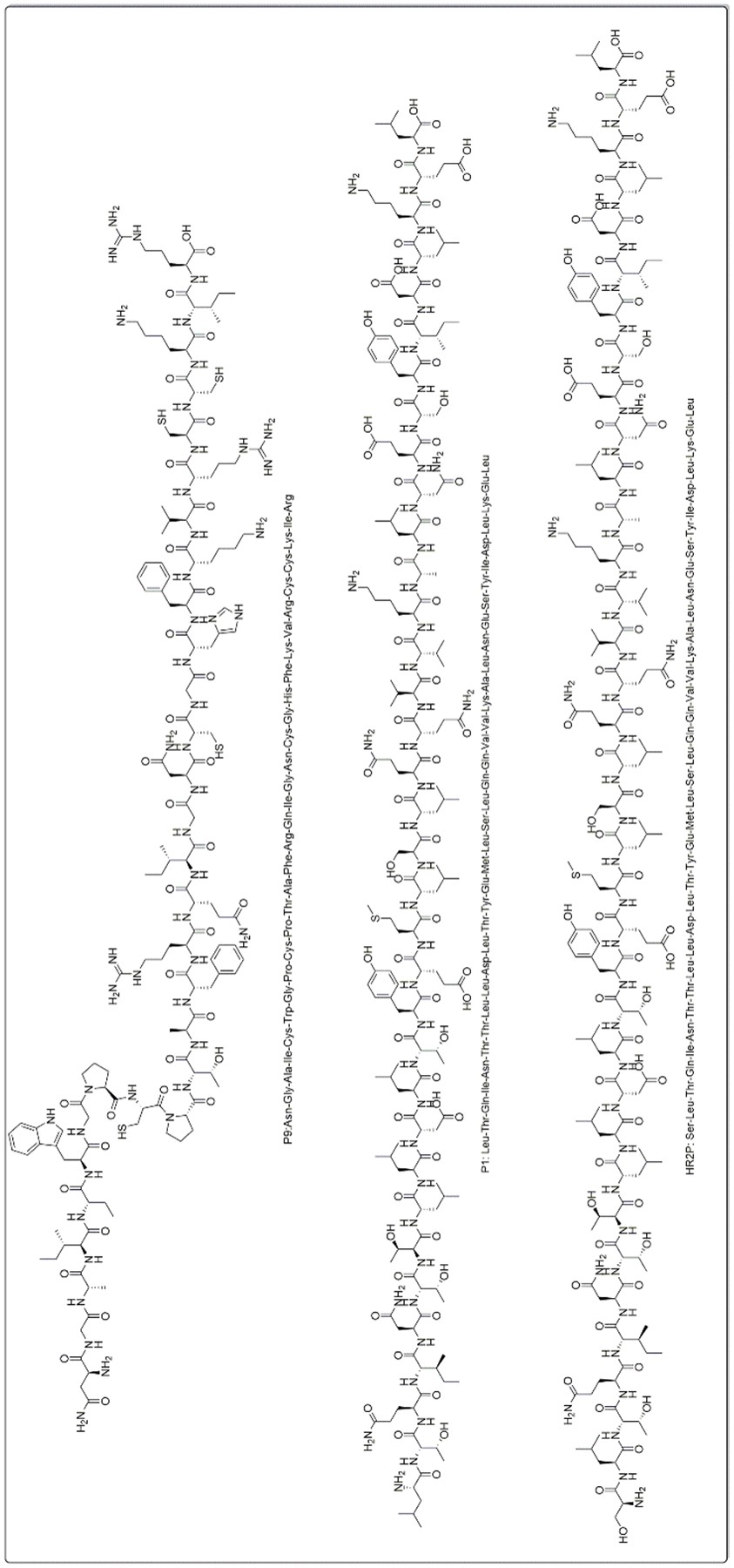

Supplement: Supplementary file 1 [file Supplementary_Figure_1.jpg]

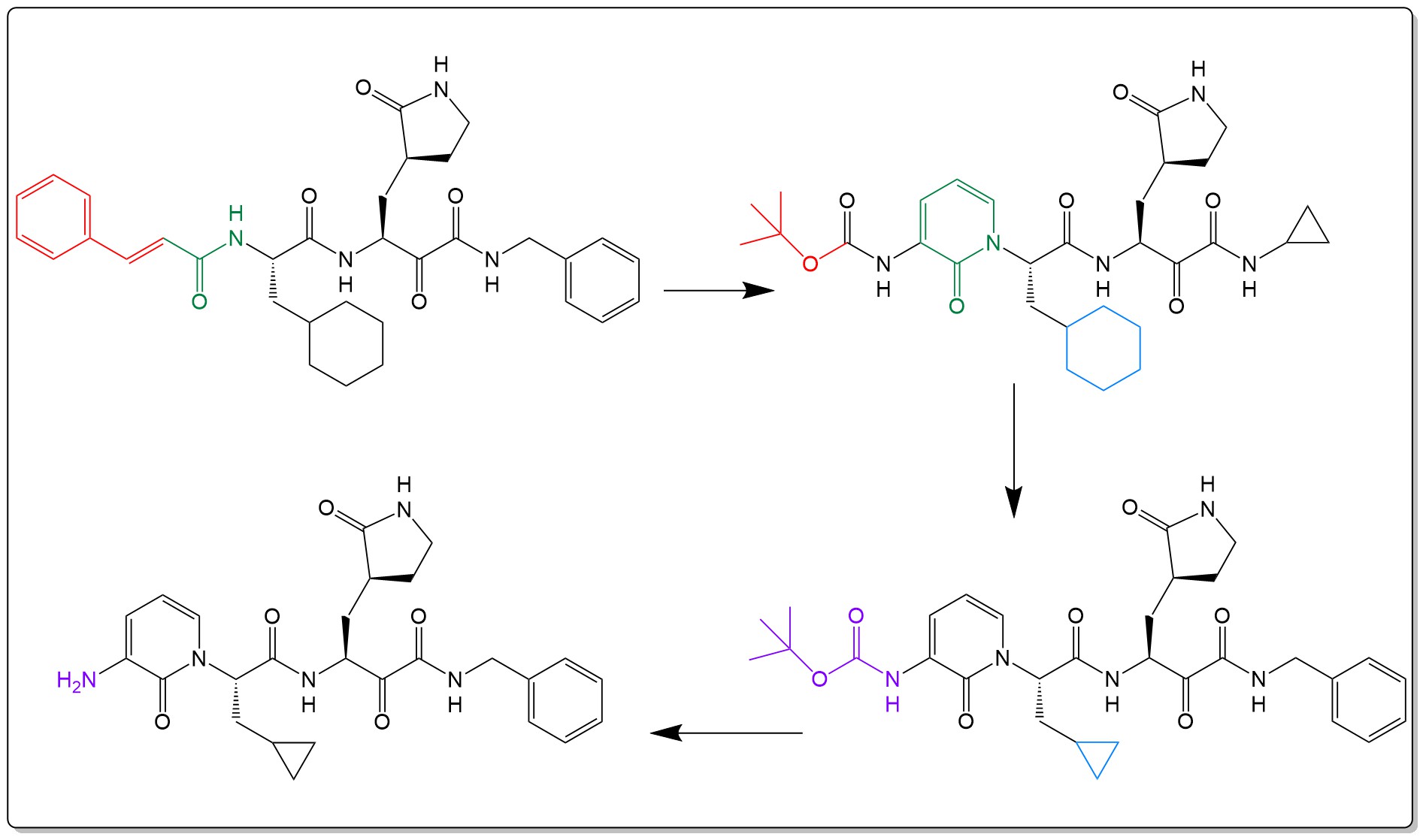

Supplement: Supplementary file 2 [file Supplementary_Figure_2.jpg]
