# Supplementary material for: Nanoscale pathogens treated with nanomaterial-like peptides: a platform technology appropriate for future pandemics
Source: Nanomedicine (Lond). 2021 May 14:10.2217/nnm-2020-0447. doi: 10.2217/nnm-2020-0447 (PMC8120868; doi:10.2217/nnm-2020-0447)
Supplement: Supplementary file 3 [file Supplementary_Tables.docx]

Table S1. Potential peptide sequences with their applications.

| Peptide | Function | Origin |
| --- | --- | --- |
| Enfuvirtide | Peptide entry inhibitors for HIV, and other viruses like parainfluenza virus, measles, and respiratory syncytial virus [65]  Same enfuvirtide/gp41 model was used to design entry inhibitors from the SARS or MERS Class I fusion protein S2 and showed the same inhibitor activity [66, 67] | Derived from the C-helix heptad repeat (CHR) domain of the subunit of the envelope protein complex of the virus (i.e., gp41) [68] |
| C34 | Peptide entry inhibitors for HIV into cells, to inhibit it and bind specifically with a pocket in gp41, therefore, prevent a conformational change for fusion | Derived from the C-heptad repeat of gp41 [69] |
| CP32M | Very potent entry inhibitor against many enfuvirtide- resistant strains of HIV | Derived from the CHR of the HIV-1 gp41 [70] |
| PsPSAPL | Significantly inhibits various bacteria growth and inhibits bacterial biofilm formation | Identified from prosaposin like sequences from starry flounder [71] |
| GFOGER | Improves bone growth and used for coating grafts | Extracted from the α1(I) CB3 fragment of type I collagen (residues from 502-507) [72, 73] |

Table S2. Examples of bifunctional peptides that act as antiviral and antimicrobial agents designed according to the above mentioned two mechanisms.

| Peptide/ Sequence | Mechanism | Source |
| --- | --- | --- |
| Magainin^[95-97]^  GIGKFLHSAKKFGKAFVGEIMNS | Virucidal | Xenopus laevis |
| Lactoferricin^[98-101]^  FKCRRWQWRMKKLGAPSITCVRRAF | Interference virus-cell surface interactions | Bos Taurus |
| Defensin (hBD-1) ^[102, 103]^  DHYNCVSSGGQCLYSACPIFTKIQGTCYRGKAKCCK | Interaction with viral glycoprotein and cell membrane | Homo sapiens |
| Mytilin-A^[104]^  GCASRCKAKCAGRRCKGWASAFRGRCYCKCFRC | Competition with the viral entry sites for binding to the cell surface | Mytilus galloprovincialis |
| Indolicidin^[105-108]^  ILPWKWPWWPWRR | Virucidal on progeny virions | Bos Taurus |
| Cecropin^[107]^  KWCFRVCYRGICYRRCR | Virucudal | Tachyplesus tridentatus |

Table S3. Amino acid composition of the receptor-binding domain of spike glycoproteins.

| Type of amino acid residues | Aromatic | Polar, uncharged | Non-polar, aliphatic |
| --- | --- | --- | --- |
|  | TYR449 | ASN487 | LEU455 |
|  | PHE456 | GLN493 | GLY496 |
|  | PHE486 | GLN498 | GLY502 |
|  | TYR489 | THR500 |  |
|  | TYR505 | ASN501 |  |
|  | TYR543 |  |  |
